# Dysmenorrhœa

**Published:** 1890-07

**Authors:** L. L. Helt

**Affiliations:** Franklin, O.


### DYSMENORRHŒA.

L. L. HELT, M. D., FRANKLIN, O.

On the fourth day of last June, at that time living in Columbus, O., was hastily called to see Miss G——, on Irving Street. The messenger, a little sister, informed me that "sister is going crazy." I at once responded to the call and found the young lady in a condition quite bewildering upon first notice. Face pale, eyes protruding, bright; labored respiration and irregular, apparently oblivious to everything around her. The mother told me she had just fainted and was "coming to." Feet and hands cold. Great thirst, only a sip at a time. Gave her Ars.$^{3x}$ in water. Whether it was efficacious or not I can't say, but believe the severity of that "storm" was past and would have done as well on placebo, as she had had those "spells" since puberty, six years ago.

In a few days she came to my office and gave the following history:

"Began menstruating at my twelfth year and have always had trouble. My old doctor used to give me morphine pills and whisky, but I kept getting worse. (How funny!)

"For three or four days before flow begins I feel as though I had rheumatism, and think every day I was coming sick.

"Severe cramps in abdomen at irregular intervals, cramps stop either first or second day of flow. Just before flow begins am *very chilly* or *very hot*. No headache. *Delirious* and *fainting* during first two days of flow; can't lie down in bed and not relieved by fanning; always in bed three or four days.

"With the delirium I always *see something alive. It may be on wall, floor, chair, any place, but it will roll upon me*, then go away, only to return, by *rolling* up on me. *It always rolls.* It changes form sometimes, but it *always rolls*. It usually has arms and small body."

Gave *Cocculus Ind.*$^{3x}$, dil. on disks, three every two hours for four days.

September 28th.—She has just passed third cycle since taking ℞ and says she has never even known what pain is since taking medicine, and is in perfect health, something she has not had for six years.

